# The aromatic dianion metalloles

**DOI:** 10.1039/c7sc04454b

**Published:** 2017-12-04

**Authors:** Junnian Wei, Wen-Xiong Zhang, Zhenfeng Xi

**Affiliations:** a Beijing National Laboratory for Molecular Sciences (BNLMS) , Key Laboratory of Bioorganic Chemistry and Molecular Engineering of Ministry of Education , College of Chemistry , Peking University , Beijing 100871 , China . Email: jnwei@foxmail.com ; Email: zfxi@pku.edu.cn

## Abstract

Metalloaromatic species are unique and important both experimentally and theoretically. Significant progress has been made during the past few decades. New aromatic systems have challenged and extended the concept of aromaticity remarkably. In this perspective, recent results on the study of the dianion aromatic metalloles and their corresponding analogues are reviewed. These include the dilithio group 14 metalloles, group 13 metalloles and transition metal metalloles. X-ray crystallography has made a key contribution to the understanding of the structures. Various theoretical tools, such as NICS and AdNDP, make it possible to measure the aromaticity beyond Hückel’s rule. The dianion butadiene skeletons play a key role in these metalloles and can be regarded as non-innocent ligands, which accept the electrons from the metal center and thus form the aromatic rings. By simply changing the central metals to different metals, the metallole analogues such as dicupra[10]annulenes and spiroaromatic palladoles can also be generated, which opens a door to synthesize other metalla-macrocyclic aromatics. Key challenges and envisioned opportunities for the future, such as applying these dianion metalloles as novel ligands of transition metals and generating new types of organometallic aromatic system, are also discussed.

## Abstract 2

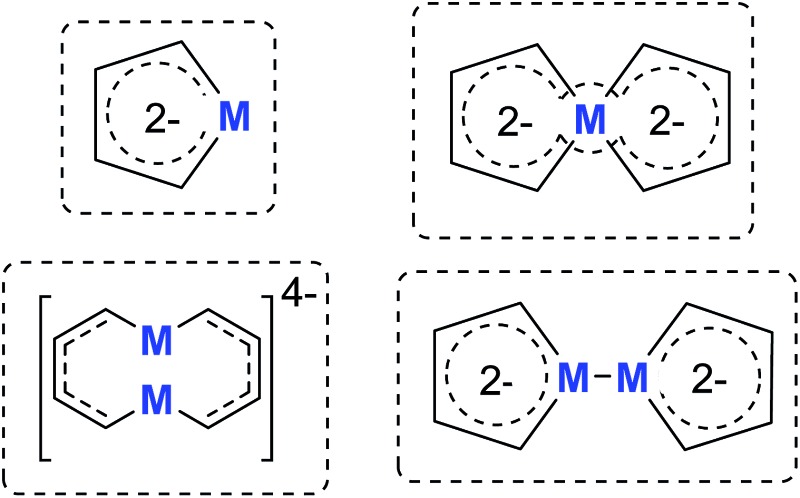
This perspective includes the synthesis and future challenges of aromatic dianion metalloles and their analogues.

## 1. Introduction

Since the isolation of benzene by Faraday in 1825,^1^ aromaticity has become a fundamental and fascinating concept in chemistry. In most organic aromatic compounds, only the π-electrons participate in delocalization. However, by introducing metals into aromatic systems, σ-, π-, δ-, and φ-electron delocalizations can all be possible,^2^ which introduces various properties to these compounds and brings a revolution to aromatic chemistry. Significant progress has been made during the past few decades in metalla-aromatics, and these new aromatic systems have challenged and extended the concept of aromaticity remarkably.^3^

Some milestones in aromatic organometallic chemistry should be noted first. In 1979, Thorn and Hoffmann predicted the existence of metallabenzenes.^4^ In 1982, the first metallabenzene, osmabenzene, was synthesized and characterized by Roper *et al.*, which opened the door of metalla-aromatic chemistry.^5^ In 2001, Jia and co-workers reported the first isolation of metallabenzyne, osmabenzyne, which is also the smallest metal carbyne.6a,b Later, Paneque *et al.* reported the first isolated metallanaphthalene, iridanaphthalene.6*c* A metallaanthracene and derived metallaanthraquinone was recently synthesized by Wright and Frogley.6*d* In 2013, Xia reported the elegant synthesis of the first metallapentalyne, osmapentalyne,^7^ which involves the d orbitals of the transition metal center in the conjugation and thus switching of the Hückel anti-aromaticity of pentalyne into the Möbius aromaticity of metallapentalyne.

Unique and useful chemical and physical properties can always be expected based on novel metalla-aromatics. Although numerous metalla-aromatics and their analogues have been synthesized, the synthesis of new types of aromatic system with different metals still remains a great challenge.

Cyclopentadiene (Cp) anions are among the most studied classic aromatic systems. By introducing metals into Cp rings, various aromatic dianion metalloles have been synthesized and characterized recently, which has opened a new page in this field. Their novel bonding models and electronic structures have attracted much attention. In this perspective, aromatic dianion metalloles and their corresponding analogues will be summarized. The remaining challenges and future opportunities in this field are also discussed at the end.

## 2. Dianion metalloles

Before discussing the aromatic dianion metalloles, several widely used measurements of aromaticity will be introduced first, as new aromatics are usually significantly different from the typical organic aromatic systems documented in textbooks. Although some aromatic systems can be judged *via* the 4*n* + 2 Hückel’s rule or the 4*n* Baird’s rule, nowadays we can use more reliable criteria which are based on quantitative measurements and calculations instead of semi-empirical and even ambiguous descriptors. This makes comprehensive analysis of aromaticity possible.

Among them, the nucleus-independent chemical shift (NICS)^8^ and its variants, the canonical molecular orbital contributions to NICS (CMO-NICS),^9^ are the most widely used theoretical indicators to judge aromaticity. Dilithio metalloles will be discussed in this perspective, and ^7^Li NMR can also be a direct experimental measurement to detect the shielding effect of the diatropic ring current.^10^ If a significant negative Li shift is observed, it means a strong shielding effect to Li atoms is present, indicating the extent of aromaticity. Moreover, energetic-based indicators such as resonance energies (REs) or isomerization stabilization energy (ISE)^11^ are also used in some aromatic systems.

Some qualitative and visual indications are also highly reliable and important, such as the adaptive natural density partitioning (AdNDP)12*a* analysis, and the anisotropy of the induced current density (ACID)12*b* analysis. They are useful and reliable tools to get theoretical insight into the nature of the delocalized bonding. With these theoretical tools in hand, the recently developed dilithio metalloles will be discussed.

### 2.1 Dilithio main group metalloles

#### 2.1.1 Dilithio metalloles of Si, Ge, Sn and Pb

It is well-known that the cyclopentadienyl (Cp) anion and its organic analogues are aromatic. However, their heavier congener analogues EC_4_^–^ (E = Si, Ge) are usually not aromatic^13^ due to the negative charge localizing on the metal center instead of the butadiene skeleton. The coordination chemistry of these EC_4_^–^ congeners was investigated by Tilley’s group in the 1990s. Interestingly, in contrast, the negative charges can be delocalized on their further reduction products, metallole dianion rings (EC_4_^2–^), which are aromatic rings.13a,^14^

**Scheme 1:**
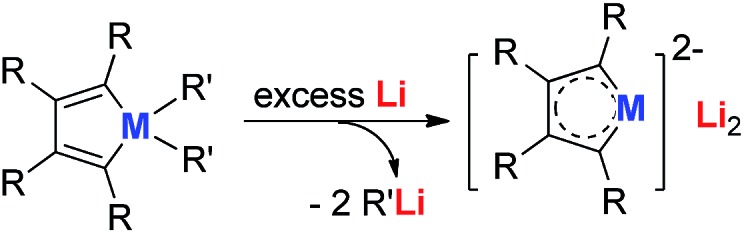
A general method to prepare the dilithio metalloles.

A straightforward way to synthesize the metallole dianion is summarized in Scheme 1. The dianion compounds can be achieved *via* a common redox reaction using the added metal lithium as the reductant to react with the corresponding metalloles.

**Scheme 2:**
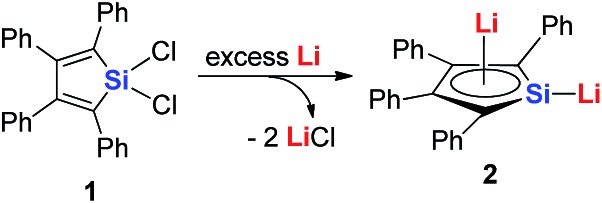
Preparation of dilithio-Si **2**.

Joo and co-workers in 1987 reported the first generation of the silole dianion C_4_Ph_4_Si^2–^.^15^ In 1995, West and co-workers reported the single crystal structure of aromatic dilithiosilole **2** (dilithio-Si).14*a* These dilithio metalloles are synthesized *via* the reduction of dichloride metalloles **1** with excess lithium (Scheme 2). The structure contains two different lithium atoms. One Li atom is bonded with the silole ring with an η^5^ fashion, while the other one is η^1^-bonded to the Si atom. The C–C bond lengths in the butadiene skeleton are averaged. The reported ^13^C and ^29^Si NMR data also supported its aromatic character.^16^

**Scheme 3:**
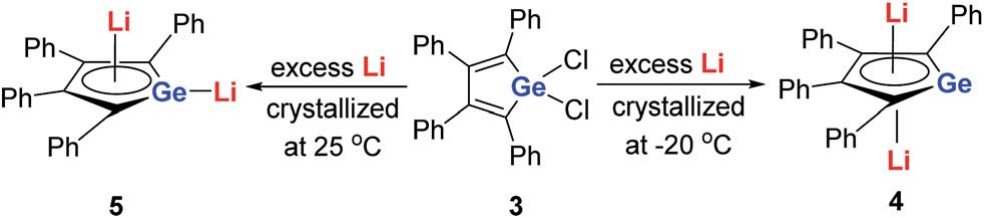
Preparation of dilithio-Ge **4** and **5**.

Later, West and co-workers reported the first characterized delocalized germole dianion with the same butadiene skeleton as dilithio-Si, the dilithio tetraphenylgermole (dilithio-Ge).14*b* Interestingly, based on their X-ray structures, the dilithio-Ge **4** generated at –20 °C has a reverse-sandwich structure, with both lithium atoms lying above and below the center of the C_4_Ge ring in an η^5^ fashion (Scheme 3), while at 25 °C, **5** was obtained instead of **4** and the two lithium atoms were bonded in an η^1^/η^5^ fashion, similar to the dilithio-Si **2**. In both cases, the C–C bond lengths in the germole ring are averaged, also pointing to delocalized π systems.

**Scheme 4:**
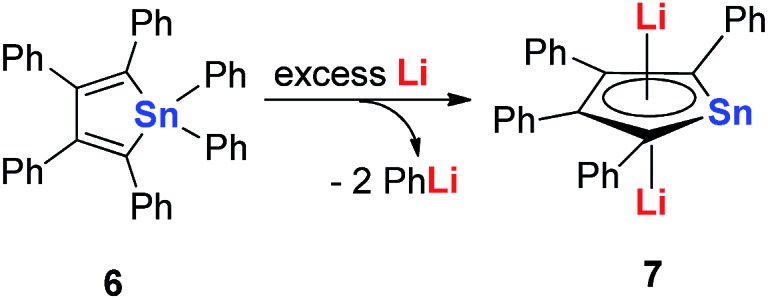
Preparation of dilithio-Sn **7**.

In 2005, Saito and co-workers reported group 14 aromatic dianion metalloles: dilithiostannole **7** (dilithio-Sn). As shown in Scheme 4, the dilithio-Sn **7** was synthesized *via* the reduction of hexaphenylstannole **6** with excess lithium in diethyl ether.^17^ The structure was confirmed by ^1^H, ^13^C, and ^119^Sn NMR spectroscopy, and X-ray analysis. Both lithium atoms are coordinated to the stannole ring in an η^5^ fashion.

**Scheme 5:**
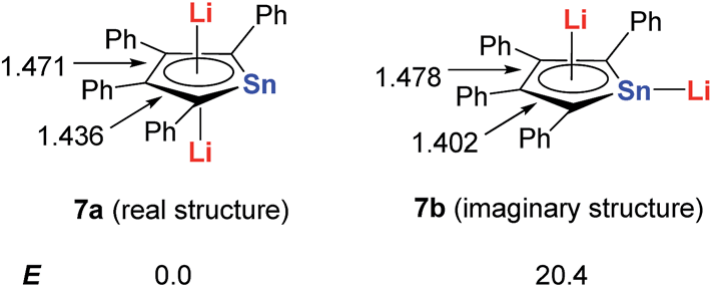
DFT calculations of dilithio-Sn **7**.

Based on DFT calculations, with Sn as the metal center, if one of the Li atoms was not bonded in an η^5^ fashion to the ring (**7b**), the energy would be 20 kcal mol^–1^ higher than that of the dilithio-Sn (**7a**) structure (Scheme 5). The bond lengths of the butadiene part in **7a** are nearly equal, while the corresponding bonds of **7b** alternate in length. These results indicate that both the central metal and Li atoms play an important role in forming the aromatic systems.

**Fig. 1:**
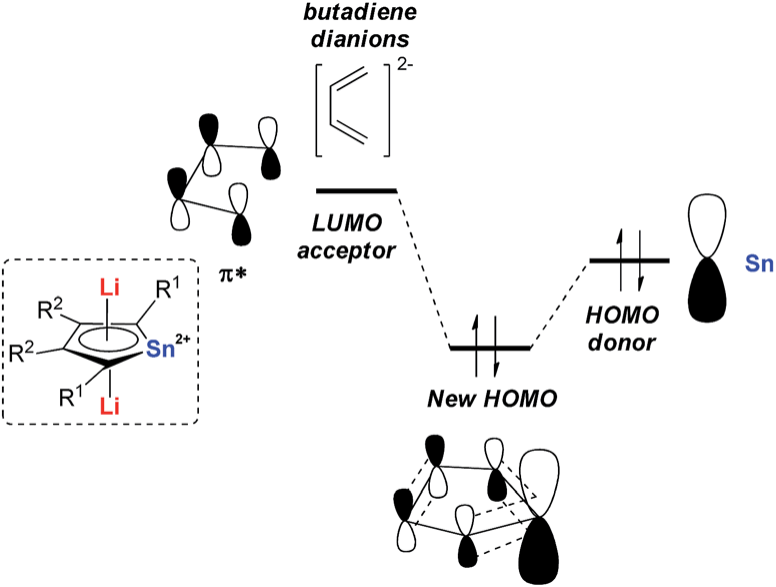
Origin of the aromatic nature of dilithio-Sn **7**.

Later in 2014, Saito and co-workers reported more detailed studies on their dilithio-Sn.^18^ Based on theoretical studies, the aromaticity originates from the delocalization of the occupied p orbitals of the metal center toward the LUMO of the butadiene part as shown in Fig. 1. Thus, the decrease of the energy gap between the two orbitals would enhance the aromaticity. In fact, the silyl-substituted stannole dianions have more stannylene character and stronger aromaticity than their corresponding alkyl- and aryl-substituted compounds, as the silyl groups attached to the butadiene moiety can stabilize (lower) the LUMO through the σ–π* conjugation. The valences of the Sn atom in dilithio-Sn are all closer to Sn(ii) than Sn(0) or Sn(iv), and were confirmed by ^119^Sn Mössbauer spectroscopy.

**Scheme 6:**
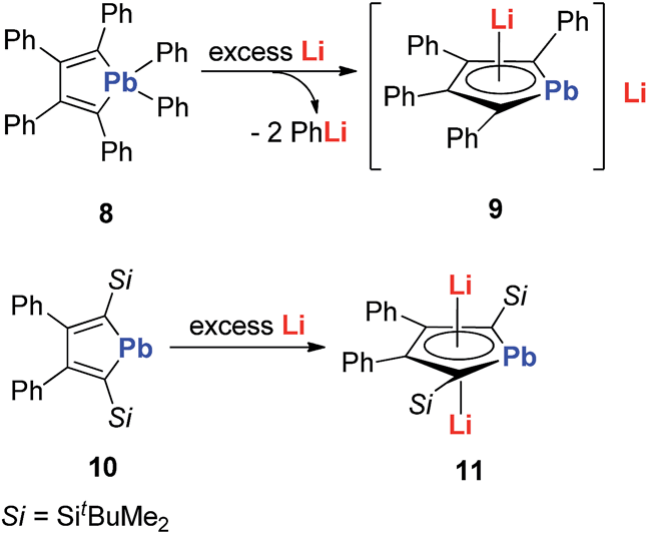
Preparation of dilithio-Pb **9** and **11**.

In 2010, Saito and co-workers reported the heaviest group 14 congener of the cyclopentadienyl (Cp) analog, dilithioplumbole **9** (dilithio-Pb) (Scheme 6).19*a* The dilithio-Pb **9** was synthesized by the reduction of hexaphenylplumbole with lithium, a similar synthetic method to that used for dilithio-Sn. Based on the single crystal X-ray structure, only one Li atom is coordinated with the plumbole ring in an η^5^ fashion, while the other Li atom is solvated and far away from the Pb center (more than 10 Å). Thus only one Li has interaction with the plumbole ring. The ^7^Li NMR spectrum has only one peak at –1.11 ppm, indicating a rapid exchange in solution between both Li atoms. The bond lengths are averaged inside the plumbole ring, suggesting that the dilithio-Pb **9** has considerable aromatic character. The remarkable negative NICS(1) value (–6.28 ppm) of free plumbole dianions also supports this conclusion.

Interestingly, in 2015 the same group reported dilithio-Pb **11** from the reduction of plumbacyclopentadienylidene **10***via* adding an excess amount of Li in toluene.19*b* Compound **11** has both Li atoms coordinated with the plumbole ring in the η^5^ fashion. **11** has a similar structure to its analogue **7**, thus it is also aromatic. And the ^7^Li NMR signal (–3.5 ppm) in C_6_D_6_ supported this conclusion. However, the ^207^Pb NMR signal for **11** (2573 ppm) shifted to a low field, compared with that for the tetraphenyl derivative **9** (1713 ppm), which indicates that its plumbylene character is enhanced by silyl groups.

#### 2.1.2 Dilithio metalloles of Al and Ga

**Scheme 7:**
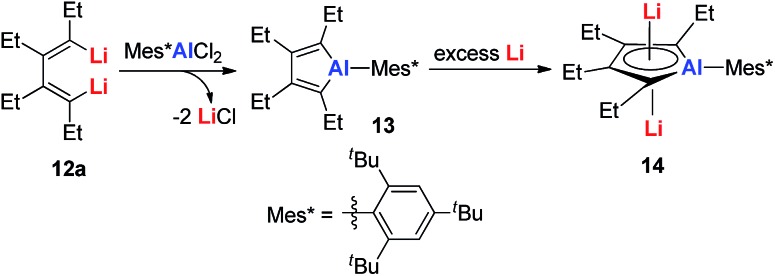
Preparation of dilithio-Al **14**.

As group 14 elements could form aromatic EC_4_^2–^ compounds, it is natural to consider whether EC_4_^2–^ complexes with other main group metals could be formed. In 2013, Tokitoh and co-workers reported dilithioalumole **14** (dilithio-Al) *via* the reduction of alumole **13** with excess Li in toluene (Scheme 7).^20^ Alumole **13** was prepared by a transmetalation reaction from the dilithio reagent **12a**.

Dilithio-Al has a similar structure to dilithio-Sn, with averaged bond lengths and two η^5^ coordinated Li atoms. As the Li atoms lie above and below the alumole core both in an η^5^ fashion, if the five-membered ring core is aromatic, significant negative shifts should be observed, because of the strong shielding effect of the diatropic ring current. The ^7^Li NMR chemical shift of dilithio-Al (–6.0 ppm) corresponds well with the calculated value (–5.5 ppm), indicating that the contact ion-pair structure is retained in solution and that the AlC_4_^2–^ skeleton is aromatic. The significant negative NICS(0) value (–15.01 ppm) also supports this conclusion.

**Scheme 8:**
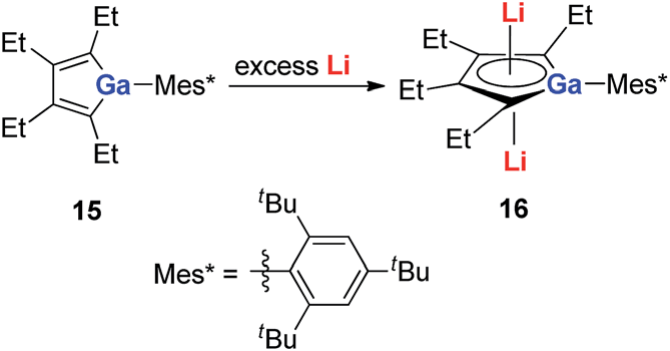
Preparation of dilithio-Ga **16**.

With a similar strategy, the dilithio-Ga **16** compounds were also synthesized by the same group in 2015 (Scheme 8).21*a* The dilithio-Ga showed almost the same structures as dilithio-Al and also showed remarkable aromaticity based on the ^7^Li NMR (–6.0 to –6.8 ppm) and NICS(1) (–15.0 to –15.5 ppm) values.

**Scheme 9:**
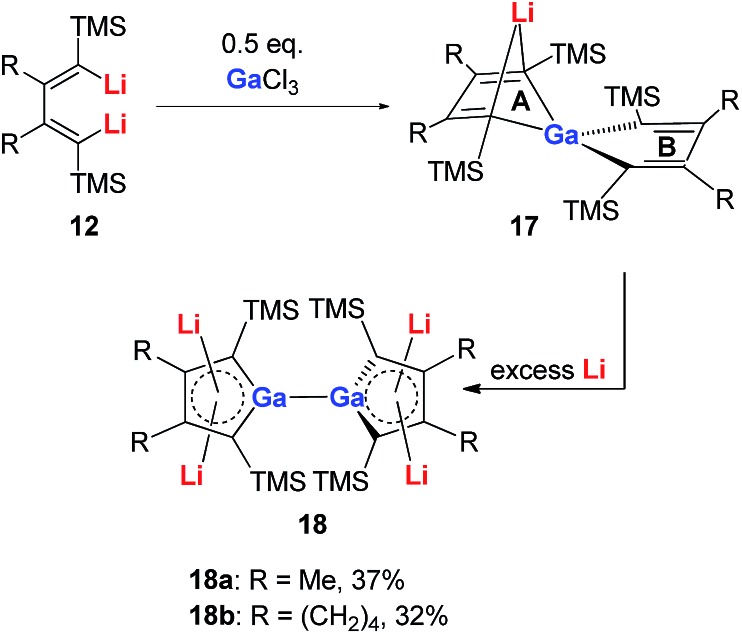
Preparation of tetralithiodigalloles **18**.

Very recently, our group reported the synthesis of aromatic tetralithiodigalloles **18** with a Ga–Ga bond *via* a similar strategy (Scheme 9).21*b* The two dilithio-Ga parts are connected with a Ga–Ga bond. Both of the dilithio-Ga units are aromatic, similar to **16**, based on the X-ray structure and ^7^Li NMR (–6.68 ppm) and NICS(1) (–11.1 ppm) values.

### 2.2 Dilithio transition metal metalloles

All the recently reported aromatic compounds contain two Li atoms and a butadiene skeleton. Their synthetic strategies were normally based on the redox reaction between an excess amount of the lithium metal and their corresponding metalloles. However, the synthesis of metalloles *via* transmetalation is in many cases impossible or extremely difficult, especially with transition metals whose corresponding salts are moderate or strong oxidizing reagents. In these cases, the corresponding aromatic dilithio metalloles could not be prepared, which limits the scope of this methodology.

To hunt for synthetic methods to create novel aromatic dilithio complexes, it is necessary to go back and re-examine the structures of the dilithio metalloles. The dilithio metalloles consist of a dilithio butadiene compound and a metal center. In fact, the LUMO of dilithio butadienes **12** is significantly lower than that of their corresponding butadienes based on our calculations. Thus, it is possible for the dilithio reagents **12** to react with appropriate low-valent metal complexes directly, providing a pair of electrons to the vacant LUMO of the dilithio skeletons.

**Fig. 2:**
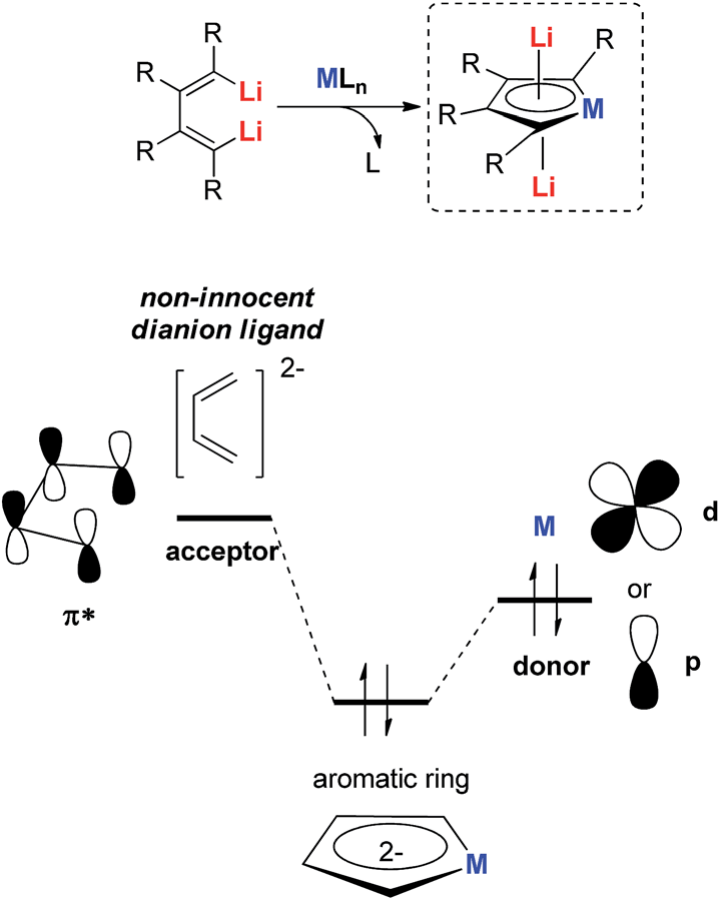
Novel method to prepare dilithio metalloles.

Our group has been working on dilithio reagents **12** for a long time^22^ and we envision that the final product could also be regarded as the combination of the conjugated dilithio butadiene part and the metal center part (Fig. 2). From this viewpoint, it is possible to synthesize various interesting metalla-aromatics readily *via* the reactions of the dilithio reagents with appropriate metal complexes.

#### 2.2.1 Dilithionickeloles (dilithio-Ni)

**Scheme 10:**
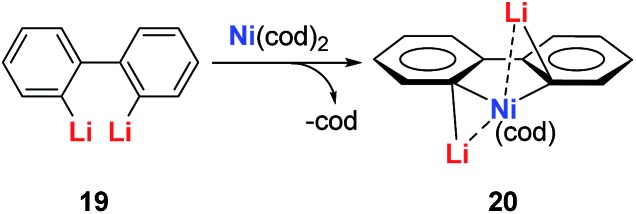
Reaction of diphenyl dilithio reagent with Ni(cod)_2_.

**Scheme 11:**
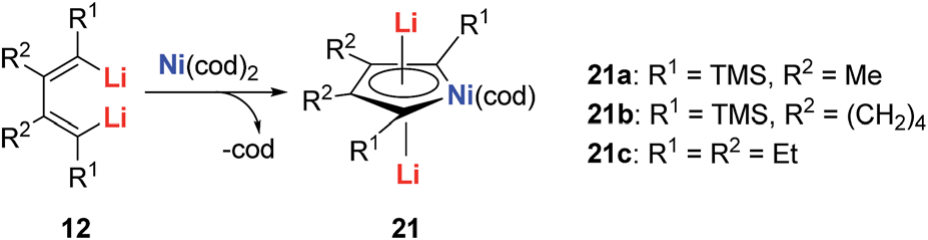
Preparation of dilithio-Ni **21**.

As reported in the literature, Ni^0^ complexes could react with stoichiometric amounts of alkyllithium reagents to afford Ni^0^ ate complexes.^23^ Similarly, taking conjugated dilithio reagents with the Ni^0^ compound Ni(cod)_2_ will offer Ni ate complexes. As expected, when the diphenyl dilithio reagent **19** was applied, the normal non-aromatic Ni(0) ate complex **20** could be isolated and characterized *via* single crystal X-ray diffraction (Scheme 10).^24^ Interestingly, when the dilithio butadiene reagent **12** was used in this reaction, the final product was aromatic dilithionickeloles **21** (dilithio-Ni), instead of the normal Ni(0) ate complexes (Scheme 11).^24^

The dilithio-Ni **21** has similar skeletons to its main group analogues (*e.g.*, dilithio-Sn). With these results, we could envision that the Ni in dilithio-Ni **21** should be Ni(ii). To confirm this hypothesis, X-ray photoelectron spectroscopy (XPS) measurements of compounds **20** and **21** were carried out. XPS of **20** detected the Ni 2p_3/2_ binding energy at 852.5 eV, falling within the range of Ni(0). To compare, XPS of **21** detected the Ni 2p_3/2_ binding energy at 855.5 eV, falling within the range of Ni(ii). This result strongly indicates that the chemical environments of the Ni atoms in **20** and **21** are clearly different, supporting the hypothesis that in compound **21**, one of the occupied orbitals of the metal center delocalizes its electron pairs toward the LUMO of the butadiene part.

Direct evidence of delocalization is that the C–C bond lengths are averaged in the skeleton of compound **21**, based on the single crystal X-ray structures, while the corresponding C–C bonds in **20** alternate in length. As expected, the ^7^Li NMR shifts in **21** are all around –6 ppm, which also strongly suggest aromaticity. To compare, the ^7^Li NMR shift in compound **20** is only –1.7 ppm. Considerable negative NICS(0) (–8.6 ppm) and NICS(1) (–10.3 ppm) values were also obtained in compound **21**, indicating aromaticity. This is also the first example of dilithio-TM (TM = transition metal) aromatic systems.

#### 2.2.2 Dilithiorhodacycle (dilithio-Rh)

To compare with group 13 metalloles (dilithio-Al and dilithio-Ga), the transition metals with valence III might also be interesting and need to be studied.

**Scheme 12:**
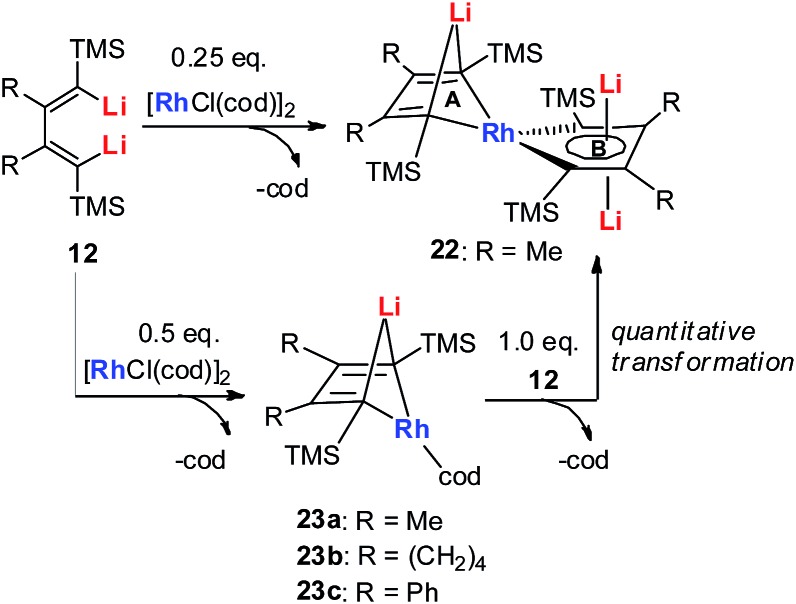
Preparation of dilithio-Rh **22**.

In 2015, our group reported the synthesis and characterization of aromatic dilithiorhodacycles **22** (dilithio-Rh).^25^ The reaction of [RhCl(cod)]_2_ with dilithio reagent **12** could offer dilithio-Rh **22** as dark red crystalline compounds (Scheme 12). In fact, this was a two-step reaction; the Rh(i) starting material first reacted with **12** to generate the rhodium ate complexes **23**, and **23** then reacted with another equivalent of dilithio reagent **12** to provide the final product **22**.

Dilithio-Rh compound **22** is a good example to show the roles of the butadiene parts. We label the two rings in **22** as **Ring A** (the Rh/Li double bridge part) and **Ring B** (five-membered dilithio rhodium ring). **Ring A** is, in fact, a normal Rh ate complex part, while **Ring B** has the metalla-aromatic structure, similar to the dilithio-M system mentioned above.

**Fig. 3:**
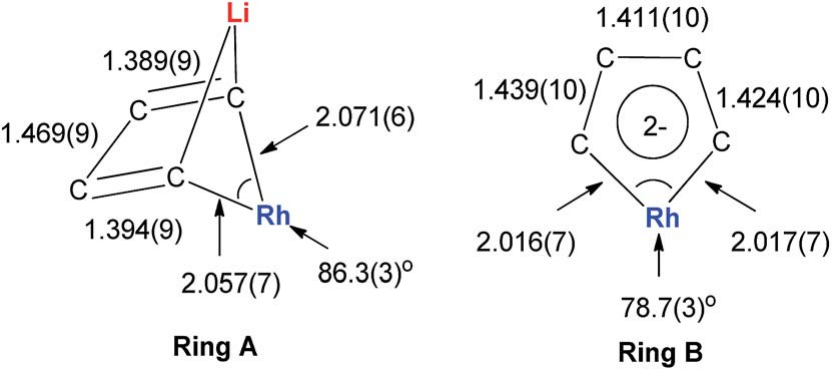
Molecular structure and selected bond lengths (Å) of **22**.

**Scheme 13:**
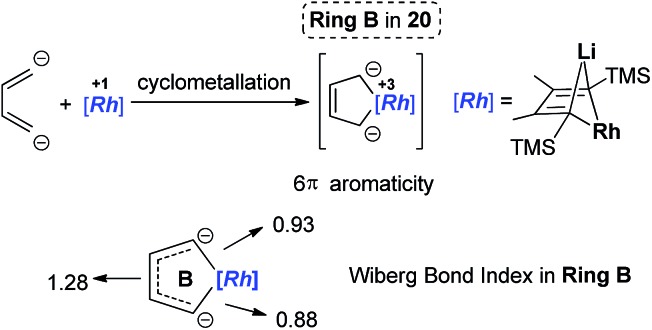
An alternative explanation of the formation of **Ring B**.

The Rh–C(sp^2^) bond lengths in **Ring B** are shorter than those in **Ring A**, implying a higher Rh–C bond order (Fig. 3). In fact, based on the Wiberg bond order, the Rh–C bond order in **Ring A** is only 0.65, while the Rh–C bond order in **Ring B** is around 0.90. Another difference is that the C–C bond lengths in **Ring A** show a clear 1,3-diene character with bond alternation, while in **Ring B** the bond lengths are averaged, suggesting a considerable delocalization effect. Thus, the formation of **Ring B** could also be explained by the cyclometallation mechanism as shown in Scheme 13.

The ^7^Li NMR spectrum also clearly shows the difference between **Ring A** and **Ring B**. The chemical shift of Li in **Ring A** is much less negative (–4.1 ppm) than that in **Ring B** (–6.3 ppm). These results also correspond well with NICS calculations (–4.6 ppm for **Ring A** and –7.8 ppm for **Ring B**).

**Scheme 14:**
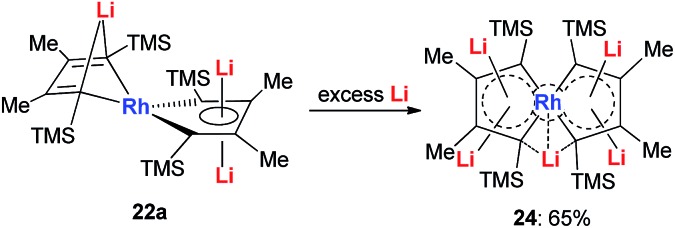
Preparation of pentalithio spiroaromatic rhodacycle **24**.

With dilithio-Rh in hand, we could further reduce this complex by reacting it with an excess amount of the Li metal in THF at room temperature.^26^ As shown in Scheme 14, the pentalithio spiroaromatic rhodacycle **24** could be obtained in 65% isolated yield and ready for X-ray single crystal analysis. One extra Li atom is located between the two Rh rings, and the corresponding Li signal in ^7^Li NMR was found at 6.37 ppm. Each of the five membered rings in the spiro rhodacycle has a similar structure to **Ring B** in dilithio-Rh.

#### 2.2.3 Tetralithio spiroaromatic palladoles

**Scheme 15:**
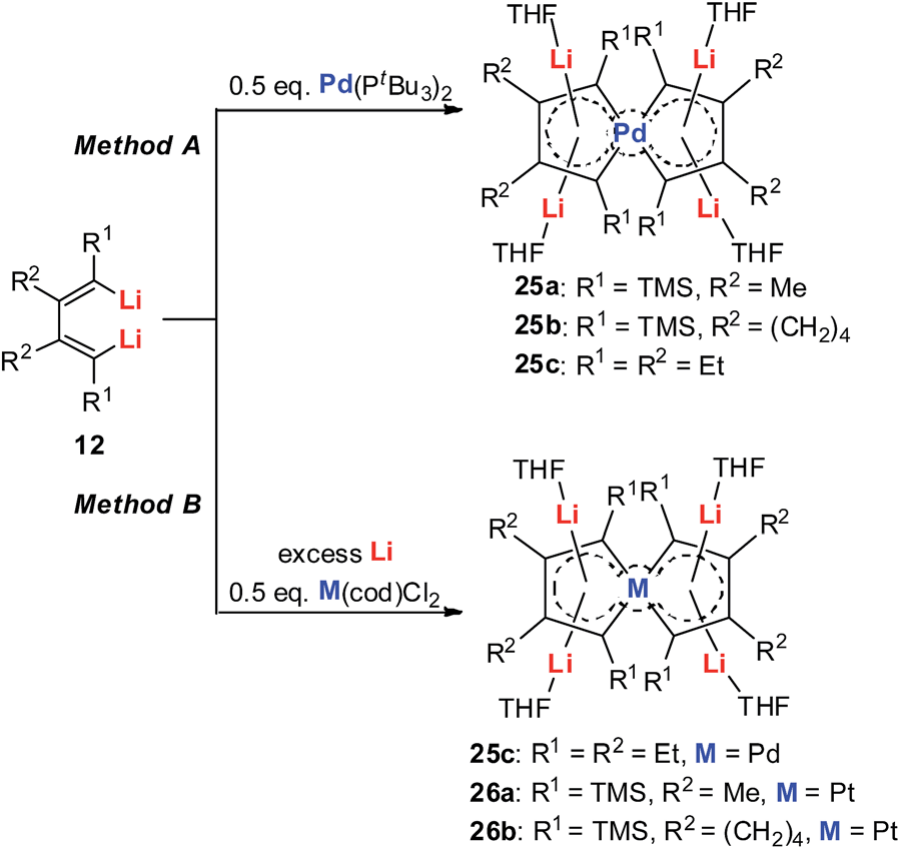
Preparation of tetralithio spiroaromatic palladoles **25** and platinacycles **26**.

Recently we reported the first series of spiro metalla-aromatics (Schemes 14 and 15).^26^ In organic compounds, based on the “tetrahedral carbon theory” by van’t Hoff, spiro-aromatic organic compounds with an sp^3^-C as the spiro atom are impossible.

Tetralithio spiroaromatic palladoles **25** could be prepared by the reaction between dilithio reagents **12** with 0.5 equivalents of Pd(P^*t*^Bu_3_)_2_ in a mixed solvent, while the tetralithio spiroaromatic platinacycles could be isolated in high yields by reacting dilithio reagents **12** with M(cod)Cl_2_ (M = Pt or Pd) in the presence of an excess amount of lithium. X-ray structural characterization shows that these spiro aromatics contain two identical metalloles that share the central metal. Each metallole is planar and has averaged C–C bond lengths. Similarly, the four Li atoms are located above and below the metallole and are bonded in the η^5^ fashion. The low frequency resonance ^7^Li NMR peaks (–4.1 to –5.2 ppm) and the corresponding NICS values (about –15.7 ppm) also support the aromaticity of these spiro metalla-aromatics.

Based on the AdNDP study, we proposed that these spiro-aromatics are 10 π-systems with two delocalized 7c-2e π-bonds on each metallole and one 13c-2e delocalized bond. The aromaticity was further confirmed by the anisotropy of the induced current density (AICD) analysis. The clockwise current density vectors indicate a diatropic ring current along the periphery of the spiropalladole ring.^26^

#### 2.2.4 Dicupra[10]annulene

**Scheme 16:**
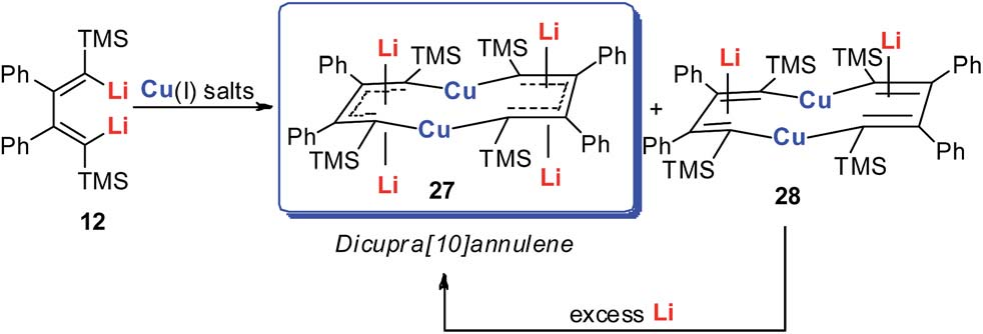
Preparation of dicupra[10]annulenes **27**.

Since the above results showed that dilithio reagents with suitable π-conjugation could be used as non-innocent ligands and novel electron acceptors, we envisioned that macrocyclic metalla-aromatics could be obtained by two or more dilithio reagents with suitable transition metals. A successful trial is shown in Scheme 16. The reaction between dilithio reagents **12** and appropriate Cu(i) salts could offer dicupra[10]annulenes **27** in moderate to good isolated yields as dark red solids.27*a*

**Fig. 4:**
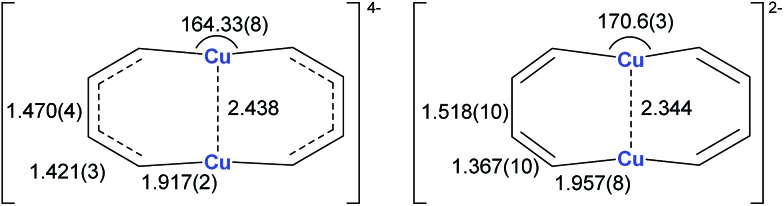
Selected bond lengths (Å) of **27** (left) and **28** (right).

Annulenes with a general formula C_2*n*_H_2*n*_ are classic cyclic conjugated systems and have been well studied. The well-known annulenes including cyclobutadiene and benzene correspond well with the Hückel rule. However, the [10]annulene that has 10 π-electrons is non-aromatic, because of the steric hindrance of the two internal hydrogens. In dicupra[10]annulenes **27**, the steric hindrance was avoided; each of the two metals could offer one electron to form delocalized π-bonds, thus it turned out to be aromatic. **27** could also be generated *via* the reduction of **28** by adding excess Li. The C–C bond lengths in **27** are averaged, whilst they alternate in the corresponding cuprate compound **28** (Fig. 4).

In fact, based on the single crystal structures, ^7^Li NMR (–5.1 to –6.2 ppm), and the NICS values (–9.8 to –12.7 ppm), there is no doubt that the dicupra[10]annulene **27** is aromatic. AdNDP suggests that there are two delocalized 8c-2e π-bonds for both half rings, together with one 14c-2e delocalized bond. Grande-Aztatzi *et al.* made a theoretical analysis of this dicupra[10]annulene system and suggested that this system could also be regarded as metalla-naphthalene.27*b* Another DFT calculation work by Zhu *et al.* suggested that the dicupra[10]annulene **27** might be 16e Craig-type Möbius aromatic, whilst compound **28** is antiaromatic.27*c*

## 3 Conclusions and outlook

In this perspective, a series of recently reported aromatic dilithio metalloles are discussed, which extend the concept of aromaticity in organometallic chemistry. However, there are still some questions and challenges remaining in this field.

The first question is whether the Li atoms are essential or not. Based on the results above, the lithium atoms play an important role on the aromaticity and should not be regarded only as the counter ion.27*c* If other alkali metals, transition metals, or even some organic cations could be introduced as the counter ion parts, then the aromatic dianion metalloles would be used as ligands, which should have a wide range of applications.

The other question is whether other types of aromatic system can be obtained by utilizing different types of the central metal. As shown in this perspective, although most of the dianion systems have similar dilithio metallole cores, a novel dicupra[10]annulene could be generated by simply changing the metals to Cu. By changing the metal center from Ni to Pd and Pt, spiro metalla-aromatics could be observed instead of the dilithio metalloles. Until now, only metals from limited groups have been introduced into this system, yet other types of metalla-aromatics should also be possible and need to be studied.

**Scheme 17:**
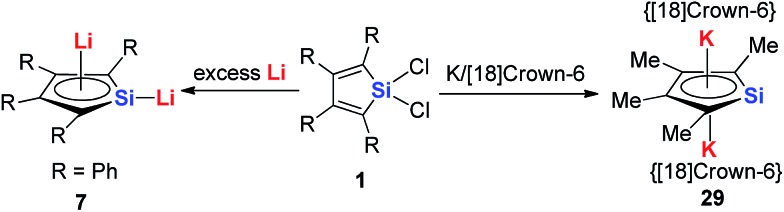
The comparison of the synthesis of the di-Li and di-K silole dianion complexes.

In regards to the first question, as the p orbitals of the Li atoms make significant contributions to the HOMO of these aromatic systems based on theoretical calculations, we expect that other alkali metals (Na, K and Cs) will have weaker interactions with the butadiene part than Li and decrease the aromaticity. In fact, there are some limited examples for the synthesis of these aromatics systems with other alkali metals. Tilley and co-workers reported the aromatic dianion silole **29** with [K([18]crown-6)^+^]_2_ as the cation parts 20 years ago (Scheme 17).13*a* Unfortunately, there is no further work reported on this system.

Other main group metals are relatively hard to introduce into this system. However, the two negative charges of the metallole might enable the coordination to transition metals. The coordination chemistry of some typical metallole anions has been investigated by Tilley’s group.^28^ Thus, it is possible to regard all the above mentioned new aromatic systems as dianion ligands of transition metals.

**Scheme 18:**
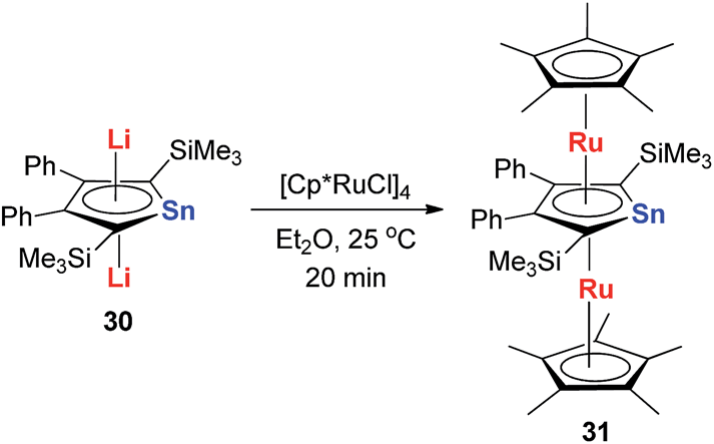
Synthesis of μ–η^5^:η^5^-stannole dianion complexes **31**.

Recently Saito and co-workers successfully replaced the Li atoms with Ru *via* transmetalation using CpRuCl (Scheme 18).^29^ The di-Ru products are still aromatic and the Ru atoms coordinate with the metallole both in an η^5^ fashion. Based on the orbital analysis, the HOMO – 2 shows that the stannole dianion moiety coordinates the ruthenium atoms as an allyl anion, while the p(Sn) overlaps with d(Ru) *via* the HOMO – 7. The silyl groups on the dilithio part might also play an important role to form these μ–η^5^:η^5^-fashions, as only the dilithio-Sn with silyl-substituted alpha-carbons can form this triple-decker structure.

**Scheme 19:**
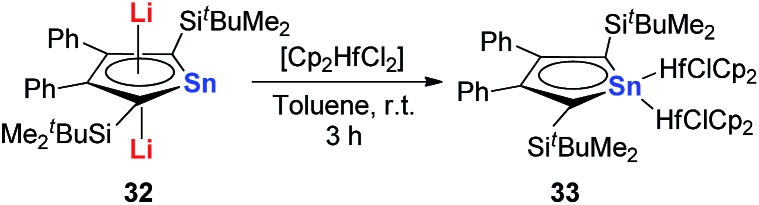
Reaction of dilithiostannole **32** with 2 equivalents of Cp_2_HfCl_2_ in toluene.

However, interestingly, when CpHfCl_2_ is used in the same transmetalation reaction, an η^1^ coordination fashion could be observed instead of the triple-decker structure (Scheme 19). As expected, remarkable C–C bond alternation was found in the butadiene part in **33**, indicating the loss of aromaticity.^30^ These examples suggest that the electronic nature of these metallole dianions is highly dependent on the coordination mode and the counter ions.

In regards to the second question, although studies of the transmetalation of the dilithio-M have been limited until now, the products of the transmetalation, especially the triple-decker aromatic complexes, could be generated *via* other strategies. These results may lead to further syntheses of other kinds of metalla-aromatics.

**Scheme 20:**
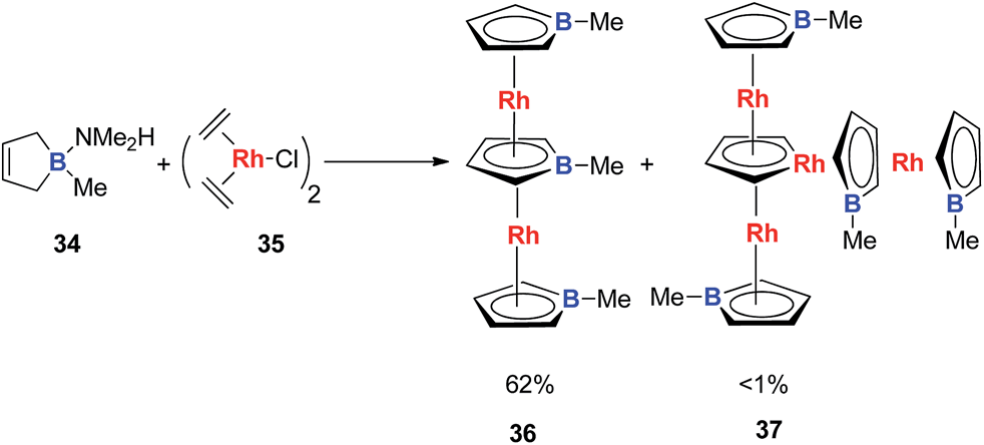
Reaction of C_4_H_6_BMe·NMe_2_H with [(C_2_H_4_)_2_RhCl]_2_.

Very recently, Loginov and co-workers reported the synthesis and characterizations of two impressive triple-decker structures **36** and **37** (Scheme 20).^31^ In particular for compound **37**, although the authors did not realize that it was an aromatic rhodacycle dianion, it should have similar electronic structures to the dianion metalloles mentioned in this perspective based on the average C–C bond lengths in the C_4_H_4_Rh ring measured by single X-ray analysis.

**Scheme 21:**
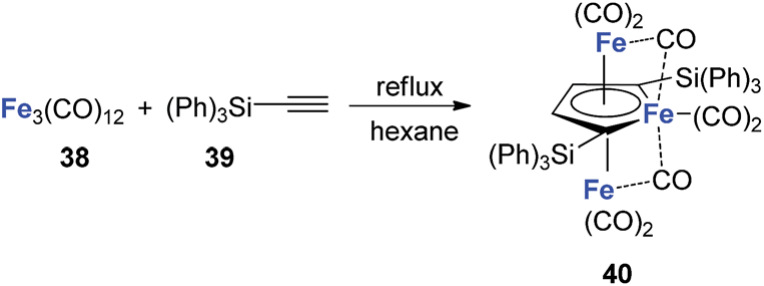
Reaction of Fe_3_(CO)_12_ with HC≡CSi(Ph)_3_.

Although the dilithio-Fe has not been synthesized yet, its triple-decker analogue has been reported and is well-studied.^32^ As shown in Scheme 21, the final product **40** has almost equal C–C bond lengths in the ferracycle. Thus it might also be regarded as an analogue of dianion metalloles. This result indicates that other kinds of dianion ferracycle are also possible.

**Scheme 22:**
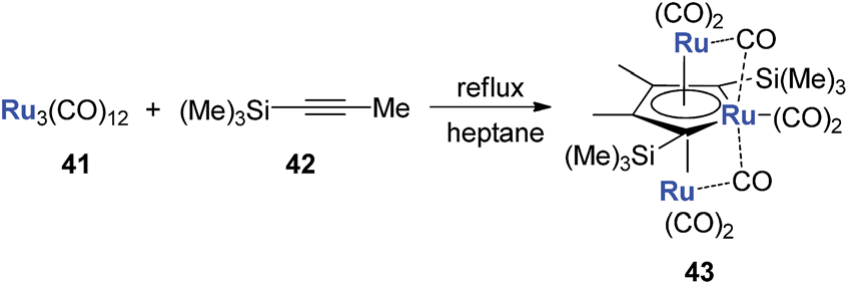
Reaction of Ru_3_(CO)_12_ with MeC≡CSi(Me)_3_.

Another example is with Ru. Following a similar strategy to that with Fe, the triple-decker ruthenacycle **43** could be prepared (Scheme 22).^33^

**Scheme 23:**
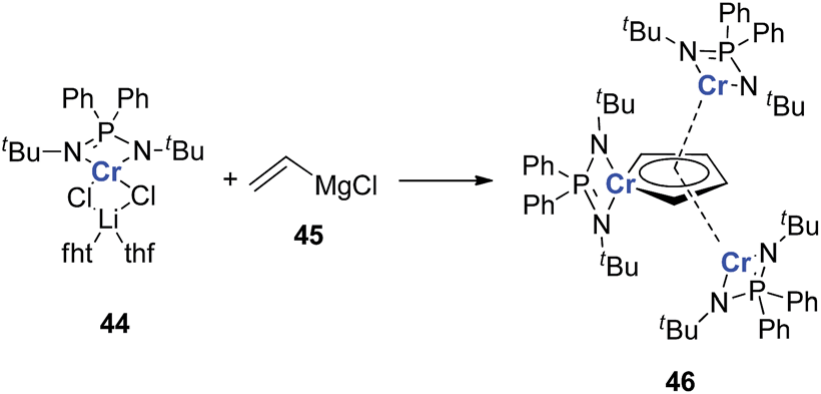
Generation of chromium complex **46**.

It should also be noted that all the examples provided in this perspective focus on the main group and late transition metals; the appropriate earlier transition metals might also be able to form dianion metalloles or other different aromatic structures. As there are various transition metals with variable valency and atomic radius, new types of aromatic system could be expected. In 2011, Murugesu and co-workers reported a novel chromium complex **46** (Scheme 23).^34^ The starting material **44** is a typical Cr(ii) lithium ate complex. Interestingly, based on their DFT calculations, all the three Cr atoms in the final product **46** are remaining Cr(ii). And the center Cr five membered ring could be regarded as a [(C_4_H_4_)Cr(L)]^2–^ species. Additionally, the C–C bond lengths in the (C_4_H_4_)Cr(L) ring are also averaged. These results are similar to the dianion metalloles mentioned in this perspective, which indicates that it is possible to generate more kinds of aromatic metallole with early transition metals.

From the works shown in this perspective, it becomes apparent that aromaticity is an attractive topic in chemistry even after decades of studies. Nowadays, the aromaticity of metalloaromatic species can be judged by reliable theoretical measurements such as NICS and AdNDP. However, a few things still remain unclear in many situations. For instance, it could be argued that the dilithio metalloles should be regarded as spherical aromatics instead of MC_4_^2–^ planar aromatics. In this case, future works need to be done to better understand aromaticity and help in the design of new compounds.

## Conflicts of interest

There are no conflicts to declare.
